# Induction of Autophagy by Cystatin C: A Mechanism That Protects Murine Primary Cortical Neurons and Neuronal Cell Lines

**DOI:** 10.1371/journal.pone.0009819

**Published:** 2010-03-23

**Authors:** Belen Tizon, Susmita Sahoo, Haung Yu, Sebastien Gauthier, Asok R. Kumar, Panaiyur Mohan, Matthew Figliola, Monika Pawlik, Anders Grubb, Yasuo Uchiyama, Urmi Bandyopadhyay, Ana Maria Cuervo, Ralph A. Nixon, Efrat Levy

**Affiliations:** 1 Nathan S. Kline Institute, Orangeburg, New York, United States of America; 2 Department of Psychiatry, New York University School of Medicine, New York, New York, United States of America; 3 Department of Pharmacology, New York University School of Medicine, New York, New York, United States of America; 4 Department of Cell Biology, New York University School of Medicine, New York, New York, United States of America; 5 Department of Pathology, Taub Institute, Columbia University, New York, New York, United States of America; 6 Department of Clinical Chemistry, University Hospital, Lund, Sweden; 7 Department of Cell Biology and Neuroscience, Juntendo University Graduate School of Medicine, Bunkyo-ku, Tokyo, Japan; 8 Department of Developmental and Molecular Biology and Medicine, Albert Einstein College of Medicine, Bronx, New York, United States of America; Mental Health Research Institute of Victoria, Australia

## Abstract

Cystatin C (CysC) expression in the brain is elevated in human patients with epilepsy, in animal models of neurodegenerative conditions, and in response to injury, but whether up-regulated CysC expression is a manifestation of neurodegeneration or a cellular repair response is not understood. This study demonstrates that human CysC is neuroprotective in cultures exposed to cytotoxic challenges, including nutritional-deprivation, colchicine, staurosporine, and oxidative stress. While CysC is a cysteine protease inhibitor, cathepsin B inhibition was not required for the neuroprotective action of CysC. Cells responded to CysC by inducing fully functional autophagy via the mTOR pathway, leading to enhanced proteolytic clearance of autophagy substrates by lysosomes. Neuroprotective effects of CysC were prevented by inhibiting autophagy with *beclin 1* siRNA or 3-methyladenine. Our findings show that CysC plays a protective role under conditions of neuronal challenge by inducing autophagy via mTOR inhibition and are consistent with CysC being neuroprotective in neurodegenerative diseases. Thus, modulation of CysC expression has therapeutic implications for stroke, Alzheimer's disease, and other neurodegenerative disorders.

## Introduction

CysC [1] is considered an important endogenous inhibitor of cysteine protease activity because of its potent *in vitro* inhibition of cathepsins B, H, K, L and S and its presence in all mammalian body fluids and tissues (for review [2]). It has a broad spectrum of biological roles in numerous cellular systems, with growth-promoting activity, inflammation down-regulating function, and anti-viral and anti-bacterial properties (for review [3]). It is involved in numerous and varied processes such as cancer, renal diseases, diabetes, epilepsy and neurodegenerative diseases such as Alzheimer's disease (AD). Its function in the brain is unclear but it has been implicated in both the processes of neuronal degeneration and nervous system repair. Enhanced CysC expression occurs in human patients with epilepsy, in animal models of neurodegenerative conditions, and in response to injury, including facial nerve axotomy, noxious input to the sensory spinal cord, perforant path transections, hypophysectomy, transient forebrain ischemia, and induction of epilepsy (for review [3]). It has been suggested that this upregulation of CysC expression in response to injury represents an intrinsic neuroprotective mechanism that may counteract progression of the disease. *In vitro* studies using various cell types exposed to a variety of toxic stimuli have reached conflicting conclusions as to whether CysC is protective or toxic to the cells (for review [3]).

The primary structure of CysC is indicative of a secreted protein and accordingly, it has been demonstrated that most of the CysC is targeted extracellularly via the secretory pathway and is taken up by cells (for review [3]). Therefore, we have studied the effect of exogenously applied human CysC on cells of neuronal origin under neurotoxic stimuli, and show here that CysC protects neuronal cells from death in a concentration dependent manner. Moreover, primary cortical neurons isolated from brains of CysC overexpressing transgenic mice [4] are more protected from death, and cells isolated from CysC knockout mice [5] are more sensitive to *in vitro* toxicity compared to cells isolated from brains of wild type mice. Our results show that the mechanism of protection does not involve inhibition of cysteine proteases such as cathepsin B. Using multiple methods, we demonstrate that CysC induces autophagy in cells under basal conditions, and enhances the autophagic activation in cells exposed to nutritional deprivation and oxidative stress.

Autophagy usually occurs in normal cells to maintain cellular turnover, and is greatly increased in cells under pathological conditions that cause cell dysfunction such as trophic stress, nutritional deprivation, hypoxia, and ischemia [6]. Macroautophagy (hereafter referred to as autophagy) is the most abundant type of autophagy that mediates sequestration and turnover of organelles and cytoplasm. Its activation reduces the size of cells and thereby decreases their metabolic burden, while generating new substrates for energy and cellular remodeling [7], [8]. During autophagy a region of cytoplasm and organelles is sequestered by a membrane that is created mainly from endoplasmic reticulum under the direction of multiple proteins, including the microtubule-associated protein MAP LC3-II, leading to the formation of a double-membrane-limited autophagic vacuole (AV) or autophagosome [9]–[11]. Autophagosomes mature to single membrane autophagolysosomes [12]–[14] and become autolysosomes by fusing with lysosomes [13]. Fusion of endosomes with autophagosomes generates amphisomes, which are subsequently cleared by lysosomes [15], [16]. The term AVs is used to refer to any of these compartments of the autophagic pathway, except lysosomes. Autophagy induction may protect cells from apoptosis by eliminating damaged mitochondria and other organelles that have the potential to trigger apoptosis [17]–[20]. However, sustained over-activity or dysfunction of the autophagic pathway in pathologic states may mediate a caspase-independent form of cell death that shares certain features with apoptosis [21]–[25].

The data presented here show that CysC induces autophagy in neuronal cells in culture under basal conditions. Cells respond to nutrition deprivation and to oxidative stress by inducing autophagy as a protective mechanism although the level of induction is not sufficient to maintain the survival of the cells. We show here, however, that under stress conditions, increased CysC levels hyper activate autophagy by further suppressing mTOR activity. CysC-induced autophagy results in enhanced degradation of long-lived proteins, which is directly related to neuroprotection. This is the first demonstration, to our knowledge, that CysC activates autophagy as a mechanism of cellular protection.

## Results

### Neuroprotection by CysC

We studied the role of CysC in the response to nutritional deprivation in mouse neuroblastoma N2a cells incubated for 48 hours in serum-free medium in the absence or presence of different concentrations of human CysC. The CysC concentrations used in this study are within the range of physiological concentrations of the protein in humans (less than 0.075 µM in serum and 0.135–0.693 µM in the CSF in subjects without apparent disorders) [26].

**Figure 1:**
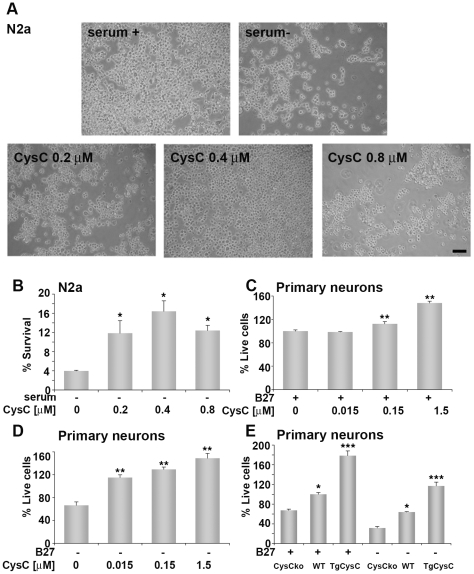
In vitro neuroprotection by either extracellular or endogenouse human CysC. **A.** Light microscopy pictures of N2a cells incubated for 48 hours in medium containing serum or in serum-free medium in the absence or presence of different concentrations of CysC. Scale bar represents 100 µm. **B.** Neuronal survival was measured by counting live cells, and expressed as percentage of neuronal survival in cultures incubated in serum-containing medium. Data are the mean and SEM of 4 experiments. **C. D.** Primary rat cortical neurons were cultured in neurobasal medium in the presence (**C**) or absence (**D**) of B27-supplement and different concentrations of human CysC for 24 hours. Cell survival analyzed by the MTS assay is expressed as percentage of live cells in cultures incubated in B27-supplemented medium without CysC. Data are the mean and SEM of 3 experiments. F and P values determined by one way ANOVA for (C) were 85.09 and <0.0001 and for (D) 34.00 and <0.0001. **E** Primary cortical neurons isolated from brains of CysC knockout (CysCko), transgenic mice overexpressing human CysC (CysCtg), or wild type (WT) mice were incubated in B27-supplement containing or lacking media. Survival is expressed as percentage of live cells in wild type cultures incubated in B27-supplemented medium. For groups incubated with B27 the F and P values determined by one way ANOVA were 20.60 and 0.0007 and for groups incubated without B27 were 68.93 and <0.0001.

Phase microscopy showed that serum-deprivation causes N2a cell-death and that the addition of CysC to the culture medium protects the cells in a concentration-dependent manner (Fig. 1A). Quantification of the effect of CysC on cell-death induced by serum-deprivation was performed by counting Hoechst nuclear stained live cells (Fig. 1B). These data confirmed the visual observations and showed that the effect of CysC on serum-deprived neuronal cells is concentration dependent, as concentrations of 0.15 to 0.4 µM CysC yielded dose-dependent increases in the survival of serum-deprived neuronal cells and CysC concentration of 0.8 µM had a lesser protective effect than 0.4 µM (Fig. 1B).

CysC also protected rat primary cortical neurons from either spontaneous death induced by culturing procedures (Fig. 1C) or more markedly from B27-supplement deprivation (Fig. 1D). The cell survival quantification MTS assay was used to quantify survival of rat primary cortical neuronal cultures incubated for 24 hours in B27-supplemented or B27-free neurobasal medium and different concentrations of CysC. The same results were obtained by quantification of survival using the Hoechst nuclear staining assay (data not shown).

Moreover, overexpression of human CysC in primary cortical neurons increased their resistance to toxicity induced by either culturing or by deprivation of B27-supplement. Mouse primary cortical neurons were cultured in neurobasal medium in either the presence or absence of B27-supplement and cell survival was quantified by the MTS assay. Cortical neurons isolated from brains of CysC knockout mice were more sensitive to both *in vitro* culturing and B27-deprivation-induced toxicity compared to cells isolated from brains of wild type mice (Fig. 1E). Primary neurons extracted from brains of homozygous transgenic mice overexpressing human CysC showed higher survival levels compared to cells isolated from wild type mice (Fig. 1E). These data show that enhanced CysC expression in neuronal cells can protect them from toxicity-induced death.

**Figure 2:**
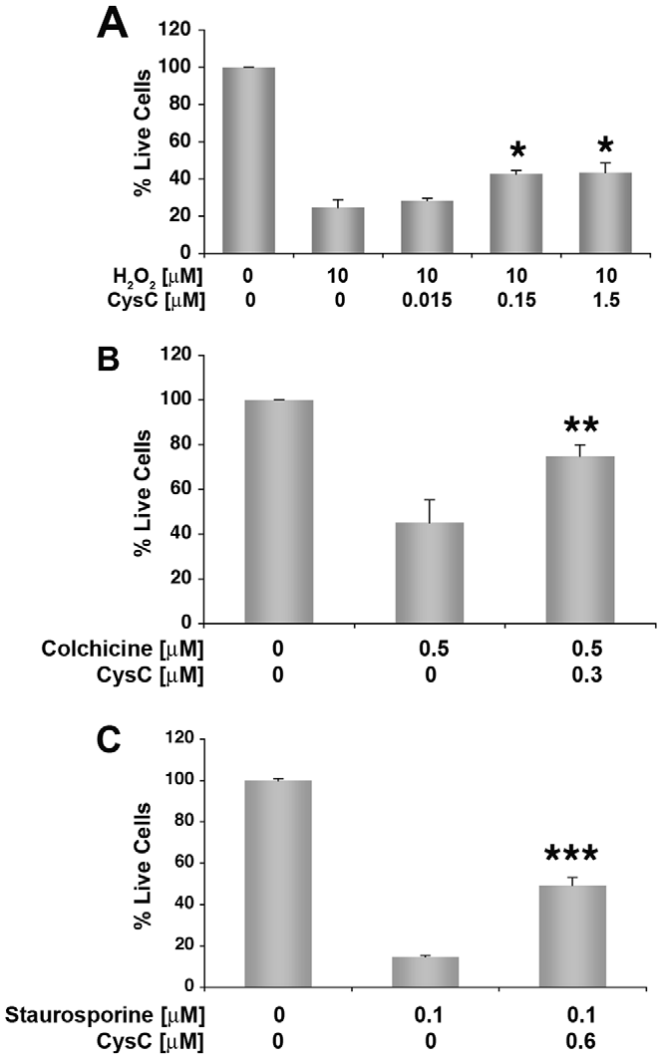
Neuroprotection of primary rat cortical neurons from a variety of insults. Primary rat cortical neurons were cultured in B27-supplemented neurobasal medium in the presence of 10 µM H_2_O_2_ (**A**), 0.5 µM colchicine (**B**), or 0.1 µM staurosporine (**C**) and different concentrations of human CysC for 24 hours. Cell survival analyzed by the MTS assay is expressed as percentage of live cells in cultures incubated in B27-supplemented medium without CysC. Data are the mean and SEM of 3 experiments. F and P values determined by one way ANOVA for (A) were 7.45 and 0.01. Differences between CysC treated and untreated samples were calculated by Student's t-test for B and C.

B27-supplement contains compounds with antioxidant activities [27], suggesting that CysC protects neuronal cells against oxidative stress. This protective effect of CysC from oxidative stress was confirmed in rat primary cortical neurons exposed to 10 µm H_2_O_2_ (Fig. 2A).

CysC also protected rat primary cortical neurons from the neurotoxic effects of a variety of other insults, including the microtubule-depolymerizing agent colchicine (0.5 µM) (Fig. 2B), and the apoptotic agent staurosporine (100 nM) (Fig. 2C).

### Neuroprotection by CysC against serum-deprivation does not involve cathepsin B inhibition

**Figure 3:**
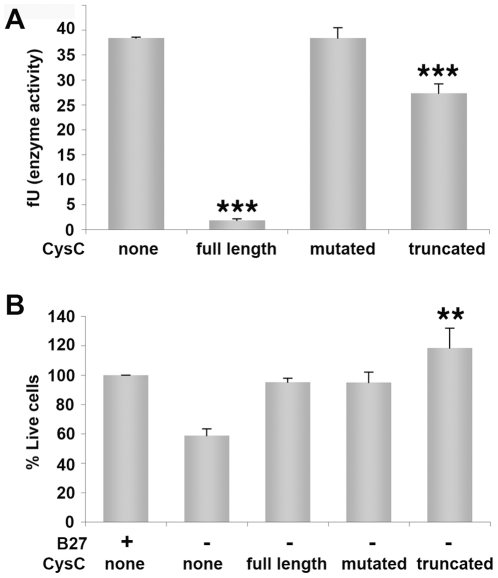
CysC forms lacking cathepsin B inhibitory activity are protective against nutrition-deprivation-induced cell death. **A**. *In vitro* analysis of cathepsin B specific activity measured in the presence of 1.7 µM of three different forms of CysC: recombinant full length human CysC; recombinant full length CysC mutated in the inhibitory active site (mutated); and N-terminally truncated human urinary CysC (truncated). Cathepsin B inhibitory activity was calculated as activity per mg of total protein and normalized for cathepsin B protein level, presented as fluorescent units (fU). Data are the mean and SEM of 3 experiments. F and P values determined by one way ANOVA were 147.6 and <0.0001. **B**. Cortical primary neurons derived from embryos of CysC knockout mice were incubated in neurobasal medium either containing or lacking B27, in the presence of 0.8 µM of the three forms of CysC. Cell survival analyzed by the MTS assay is expressed as percentage of live cells in cultures incubated in supplemented media without CysC. Data are the mean and SEM of 3 experiments. F and P values determined by one way ANOVA were 9.39 and 0.005.

In order to test the possibility that the mechanism of protection by CysC involves inhibition of cathepsin B, cathepsin B expression and specific activity were measured in neuroblastoma cells under serum-deprivation conditions in the presence or absence of CysC. Cathepsin B levels were quantified by Western blot analysis and normalized to β-tubulin levels for each condition. Cathepsin B protein levels or specific activity did not differ between cells in the presence or absence of CysC (data not shown). We then compared the protective effect of human urinary CysC, used in all the experiments described here, with that of two other forms of the protein. The CysC isolated from human urine is truncated at the N-terminus by 10 amino acids, which has been previously shown to diminish the cathepsin inhibitory activity of the protein [28]. This truncated form was compared to a recombinant full length human CysC and to a mutated recombinant full length CysC in which most of the residues in the inhibitory active site have been replaced by glycine residues, retaining its overall 3-D structure. Compared to full length wild-type CysC, this Gly-substituted variant has negligible inhibitory activity toward the mammalian cysteine peptidases, cathepsins B, H, K, L, and S [29]. Measurements of cathepsin B specific activity *in vitro* confirmed that the Gly-mutated CysC is unable to inhibit cathepsin B, and that the N-terminally truncated human urinary CysC has lowered cathepsin B inhibitory activity compared to full length human CysC (Fig. 3A). We then compared the relative protective effects of the three forms of CysC on mouse cortical primary neurons incubated in B27-containing or B27-free neurobasal media (Fig. 3B). We used cells derived from CysC knockout embryos [5] in order to specifically study the effect of the three different forms of CysC in the absence of full length endogenous CysC. Despite having markedly different inhibitory activities toward cathepsin B, all three forms of CysC protected cultured cortical neurons from death induced by B27-supplement deprivation (Fig. 3B). These data preclude cathepsin B inhibition as the mechanism of protection by CysC under the conditions tested here.

### CysC does not protect neuronal cells from toxicity when autophagy is blocked

**Figure 4:**
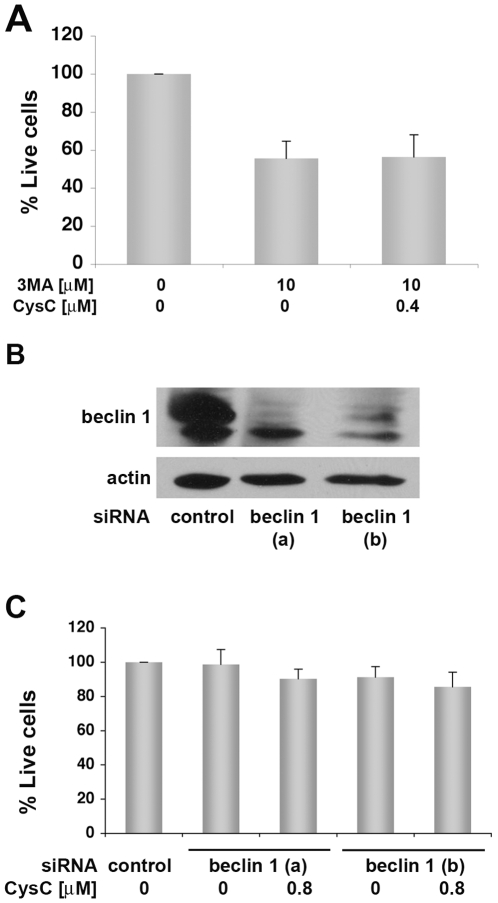
CysC does not protect neuronal cells from toxicity when autophagy is inhibited. **A.** N2a cells were incubated for 44 hours in serum-free medium with 3MA, an inhibitor of autophagy, in the presence or absence of 0.4 µM CysC. Neuronal survival was measured by counting live cells, and expressed as percentage of neuronal survival in cultures incubated in serum-deprived medium. Data are the mean and SEM of 4 experiments. There were no significant differences between samples containing 3MA with or without CysC. **B.** N2a cells were treated with two forms of *beclin 1* siRNA (a and b). Western blot analysis with anti-beclin 1 antibody shows inhibition of beclin 1 expression in treated cells. **C.** *Beclin 1* siRNA treatment of N2a cells attenuates the protective effect of CysC from serum-deprivation induced death. Cell survival was measured by the MTS assay. Data are the mean and SEM of 3 experiments. No significant difference between CysC treated and non- treated cells was observed.

Cells respond to nutritional withdrawal by inducing autophagy that is crucial for cell adaptation and survival under extreme conditions. This process allows the degradation of intracellular macromolecules and provides the energy required for minimal cell functioning when nutrients are scarce (for review [30]). We tested whether or not the protective effect of CysC is preserved under conditions in which autophagy is inhibited. CysC did not protect N2a cells from the toxicity of the autophagy inhibitor, 3-methyladenine (3MA) [31], under conditions of serum-deprivation (Fig. 4A). To further advance our understanding of the role of autophagy in the response of cells to CysC treatment, we studied the consequences of autophagy disruption by treating N2a cell cultures with two different small interfering RNA (siRNA) targeting specifically *beclin 1* mRNA. Beclin 1 is a coiled-coil, myosin-like BCL2-interacting protein, a well characterized autophagy activator [31]. The two siRNA efficiently reduced levels of beclin 1 expression in N2a cells (Fig. 4B). No significant difference was observed between N2a treated with *beclin 1* siRNA in the presence or absence of CysC (Fig. 4C). Thus, silencing of beclin 1 expression, which inhibits the autophagic process [31], eliminated the protective effect of CysC from serum deprivation-induced death (Fig. 4C).

### CysC induces autophagy

**Figure 5:**
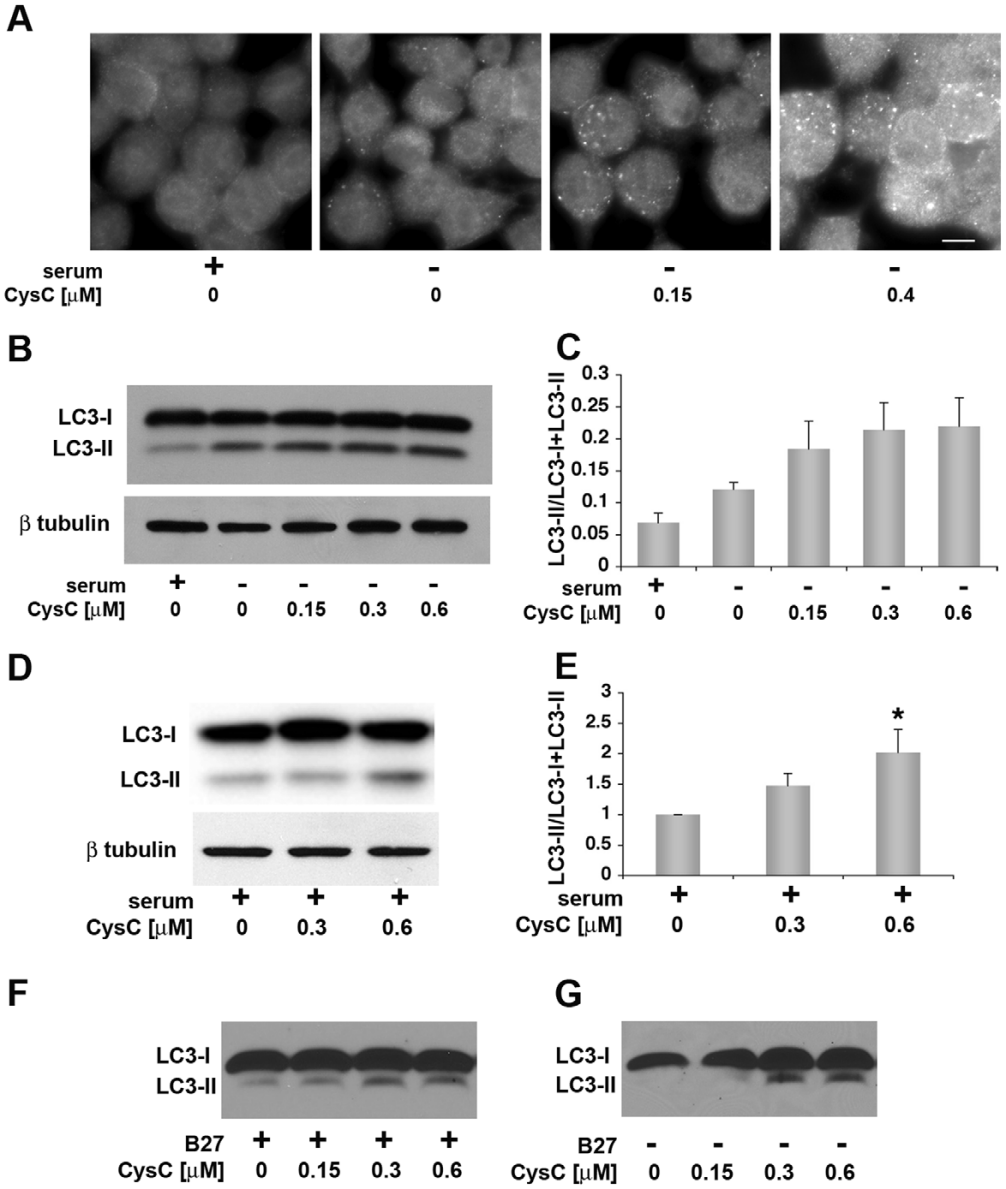
CysC induces cleavage of LC3 and the generation of vesicles stained for LC3-II, associated with autophagic vesicles. **A**. N2a neuroblastoma cells were incubated for 18 hours in serum containing or serum-free media containing the indicated concentrations of CysC. Cells were stained with an anti-LC3 antibody enriched for LC3-II and visualized in a fluorescence microscope. Scale bar represents 10 µm. **BE**. N2a cells were incubated in serum-supplemented or serum-free media containing different concentrations of CysC for 12 hours. Cell lysate proteins were analyzed by Western blot using anti-LC3 antibodies. Representative images of Western blot analyses of cell lysate proteins in serum-free medium (**B**) and in serum-supplemented medium (D) are shown. The intensity of the LC3-I and LC3-II bands was measured and results are expressed as the ratio of LC3-II to total LC3 (LC3-I + LC3-II) values (**C** and **E**). Data are the mean and SEM of 4 experiments. F and P values determined by one way ANOVA for (E) were 5.11 and 0.02 (**F** and **G**) Western blots with anti-LC3 antibodies of primary cortical neurons isolated from CysC knockout mice incubated for 12 hours in media containing (**F**) or lacking (**G**) B27-supplement in the presence of different concentrations of CysC.

LC3 is a selective marker for AVs, which appears cytosolic in normal conditions (LC3-I) and becomes punctate when it is lipidated and cleaved to form LC3-II that is associated with membranes of AVs [31]. In order to analyze the effect of CysC on the levels of LC3-labeled vesicles in serum deprived cell cultures, we measured LC3-II and LC3-I protein levels in N2a cells incubated in serum-containing or serum-free media, supplemented with various concentrations of CysC. Immunohistochemical studies using an anti-LC3-II enriched LC3 antibody indicated that addition of CysC induced a concentration-dependent increase in the number of LC3-II labeled vesicles (Fig. 5A).

In support of these data, Western blot analysis showed that CysC enhances the formation of LC3-II in serum-deprived neuronal cells (Fig. 5B). N2a neuroblastoma cells were incubated in serum-supplemented or serum-free media and the serum-free medium contained increasing concentrations of CysC. Cell lysate proteins were separated by gel-electrophoresis and blotted with anti-LC3 antibodies (Fig. 5B). Quantification of the LC3-I (16 kDa) and LC3-II (14 kDa) bands showed that N2a cells respond to serum deprivation by the formation of LC3-II, and that CysC treatment is associated with increased conversion of LC3-I into LC3-II (Fig. 5C). Addition of CysC to serum-containing medium also induced conjugation of LC3 in N2a cells (Fig. 5D and E), revealing that CysC affects basal autophagy under normal culture condition. Furthermore, CysC increased conversion of LC3-I into LC3-II in primary cortical neurons isolated from CysC knockout mice in either the presence or absence of B27-supplement (Fig. 5F and G).

**Figure 6:**
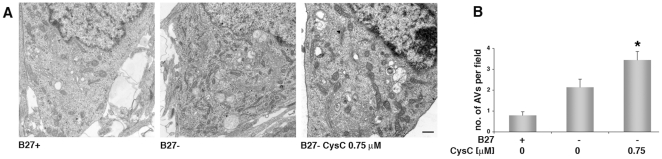
CysC induces an increase in the number of autophagic vesicles (AVs) in neuronal cells. Electron micrographs of primary rat cortical neurons (**A**) incubated for 12 hours in either B27-supplemented or deprived medium in the absence or presence of 0.75 µM CysC. The total number of AVs *per* cell was counted for at least 20 cells/condition, and the average number of vesicles *per* cell is shown (**B**). Data are the mean and SEM. Differences between CysC treated and untreated samples were calculated by Student's t-test.

In order to confirm our finding that CysC induces autophagy in neuronal cells, electron microscopy studies were conducted on rat primary cortical neurons in B27-free medium in the absence or presence of CysC (Fig. 6A). Counting the total number of AVs per cell, including autophagosomes and autophagolysosomes, confirmed that the removal of B27-supplement induced the appearance of AVs in these cells, and CysC further enhanced this process (Fig. 6B).

### CysC increases the levels of proteolysis in lysosomes partially due to autophagy in serum-deprived neuroblastoma cells

**Figure 7:**
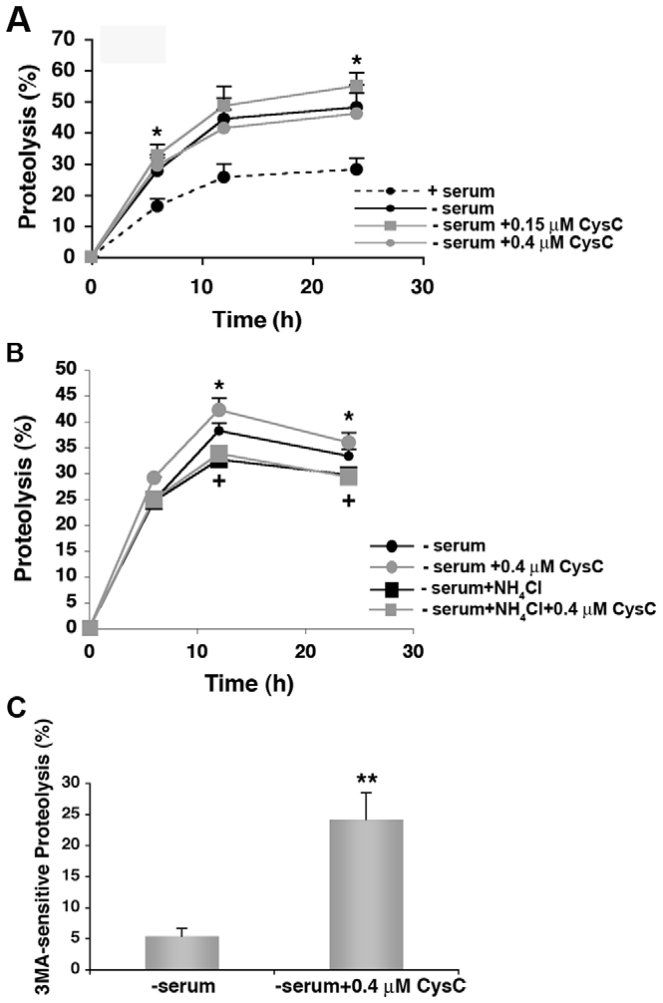
CysC enhances total lysosomal-dependent protein degradation in serum-deprived neuronal cells. **A.** Effect of increasing concentrations of CysC on total rates of protein degradation. N2a cells were labeled for 2 days with [^3^H]-leucine. After extensive washing, cells were incubated in serum-containing or serum-free media. Removal of serum maximally activates lysosomal degradation. The cells maintained in serum-free media were supplemented or not with increasing concentrations of CysC as labeled. The rate of total protein degradation at the indicated times was calculated as the percentage of total radiolabeled protein transformed into soluble amino acids. **B.** Effect of inhibition of lysosomal proteolysis on the CysC-induced increase in protein degradation. N2a cells were labeled as in A and then maintained in serum-free media and supplemented or not with CysC. Where indicated 20 mM NH_4_Cl and 100 µM leupeptin were added to inhibit lysosomal proteolysis. Protein degradation was calculated as in A. **C**. Effect of CysC on macroautophagy-dependent proteolysis. N2a cells labeled as in A and maintained in serum-free media were supplemented or not with CysC. Half of the cells were treated with 10 mM 3MA to inhibit macroautophagy. The percentage of lysosomal degradation that results from autophagic degradation (3MA sensitive), in the presence or absence of CysC was calculated. Values are mean and SED of triplicate wells in 3–4 different experiments. One way ANOVA for differences between CysC treated and untreated samples were significant for *p = 0.05; **p = 0.001 and between control and ammonium chloride treated samples were significant for **^+^** *p* = 0.01.

An increase in the number of AVs in the cell may result from either enhanced generation of the vesicles or blockage of their fusion with lysosomes. While induction of autophagy and resultant enhanced protein degradation would protect the cell from toxic insults, accumulation of AVs without their efficient clearance could contribute to cell death. In order to differentiate between the two conditions, we analyzed total protein degradation in serum-deprived neuronal cells. Quantification of the rate of total protein degradation in N2a cells showed increased proteolysis in the presence of CysC in a dose-dependent manner (Fig. 7A).

The increase in protein degradation observed upon treatment with CysC was no longer observed if lysosomal degradation was inhibited by treatment with NH_4_Cl and leupeptin, indicating that CysC stimulated protein degradation in lysosomes (Fig. 7B). Because different types of autophagy can contribute to protein degradation in lysosomes, we estimated the relative contribution of macroautophagy to the observed changes in lysosomal protein degradation, by performing similar experiments in 3MA treated cells. Macroautophagy degradation is calculated as the percentage of lysosomal degradation (sensitive to NH4Cl and leupeptine) that can be inhibited by treatment with 3MA. Analysis of the contribution of macroautophagy to the lysosomal degradation induced by serum deprivation in the presence or absence of CysC showed that CysC caused a marked shift of the lysosomal degradation towards macroautophagy (Fig. 7C). These studies confirm that the increase in the number of AVs observed in CysC treated N2a cells was the consequence of enhanced autophagic activity. These data indicate that CysC enhances the flux through fully functional autophagy, resulting in delivery of cytosolic cargo to lysosomes where degradation occurs.

### CysC induces autophagy via the mTOR pathway

**Figure 8:**
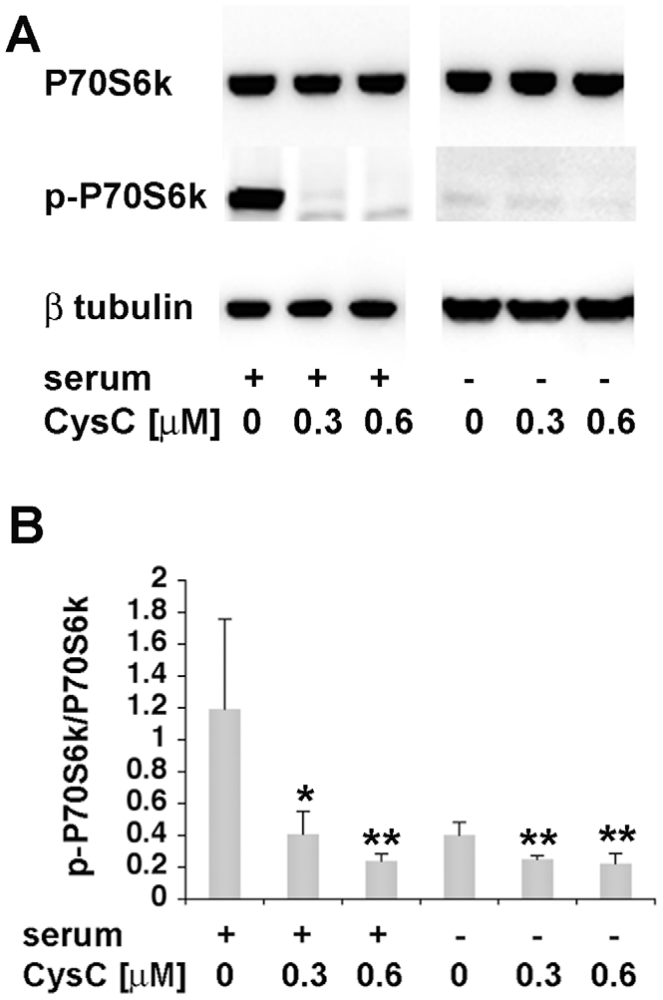
CysC induces autophagy via the mTOR signaling pathway in either serum-containing or serum-free medium. **A.** N2a cells were incubated for 12 hrs in serum-containing or serum-free medium in the presence or absence of CysC. Cell lysate proteins were separated by gel electrophoresis and blotted with antibodies to P70S6 kinase, p-P70S6 kinase (Thr389), or β-tubulin. Representative images of Western blot analysis are presented. **B.** The intensity of the bands was measured, and p-P70S6 protein levels were calculated relative to total P70S6 values showing a decrease in the level of P70S6 kinase phosphorylation. Data are the mean and SEM of 6 experiments. For serum containing groups the F and P values determined by one way ANOVA were 9.07 and 0.006 and for serum deprived groups were 10.18 and 0.005.

In order to determine the autophagic signaling pathway induced by CysC, the effect of CysC on the level of p70S6 kinase phosphorylation (p-p70S6 kinase), a substrate protein in the mTOR pathway [31], was analyzed. N2a cells were incubated in either serum-containing or serum-free medium in the absence or presence of CysC. Cell lysate proteins were separated by gel electrophoresis, transferred onto a membrane, and blotted with antibodies to either p70S6 kinase or p-p70S6 kinase (Fig. 8A). Band intensities were measured and p-p70S6 kinase levels were calculated relative to total p70S6 kinase values. While the level of p70S6 kinase was not affected by either serum deprivation or CysC (Fig 8A), the level of its phosphorylation was significantly reduced by CysC exposure in serum-containing medium (Fig. 8B). Serum-deprivation, as expected, markedly reduced p70S6 kinase phosphorylation and, importantly, a further significant reduction of p70S6 kinase phosphorylation was achieved by the addition of CysC to the serum-free medium (Fig. 8B). The phosphorylation state of p70S6 kinase is an established index of mTOR activity [32], [33], with dephosphorylation of p70S6 kinase indicating inhibition of mTOR, a negative regulator of autophagy. These data, therefore, indicate that CysC induces autophagy through inhibition of the mTOR-signaling pathway.

## Discussion

The data presented here demonstrate a protective effect of CysC on neuronal cells cultured under serum-deprivation conditions. CysC also protects primary cortical neurons against the toxicity induced by oxidative stress, the microtubule-depolymerizing agent colchicine, and staurosporine, a potent wide spectrum inhibitor of protein kinases [34]. The secreted protein [3], exogenously applied to neuronal cells, protects neuronal cells from death in a concentration dependent manner. While an increase in protection was observed for all CysC concentrations added to the culture medium of primary cortical neurons, a decrease in protection by high CysC concentrations was observed for N2a cells. These data suggest a difference in sensitivity to CysC and/or bioavailability due to variable uptake or delivery of CysC by different neuronal cell types. Moreover, endogenous CysC overexpression in primary cortical neurons isolated from brains of CysC transgenic mice [4] also protected the cells from spontaneous death induced by culturing and from B27-supplement-deprivation. Cells isolated from CysC knockout mice [5] were more sensitive to *in vitro* toxicity compared to cells isolated from brains of wild type mice.

Multiple *in vivo* and *in vitro* studies have previously demonstrated the involvement of CysC in the response to a variety of neurodegenerative insults, supporting either a protective or toxic effect of enhanced levels of CysC. Given the diverse roles that CysC plays, CysC could either contribute to a cell-death mechanism that is activated, or be protective by preventing cell death, promoting cell survival, or promoting mitogenic activity resulting in neurogenesis. The mechanisms that were previously proposed to explain CysC function either involve inhibition of cysteine proteases such as cathepsin B or are independent of cathepsin inhibition. The demonstration of *in vitro* inhibition of cysteine proteases by CysC suggested that CysC is an endogenous inhibitor of lysosomal proteinases (for review [2]). Cathepsins have been linked with cell death mechanisms and enhanced expression of several cathepsins have been documented in response to injuries similar to those inducing CysC upregulation (for review [35]). Furthermore, inhibitors of cathepsins B and L reduced neuronal damage in the hippocampus after ischemia (for review [35]). Thus, an increase in CysC expression might be a response to rescue neurons by inhibiting the apoptosis-promoting actions of cathepsins. However, CysC has a role in neuronal proliferation, differentiation, and possibly neuroregeneration, independent of its effects on cathepsin activity [36]–[40].

Leukocyte elastase in catalytic amounts was observed to rapidly cleave the Val-10-Gly-11 bond of the human CysC at neutral pH. The resulting truncated CysC had size and amino acid composition consistent with a CysC molecule devoid of the N-terminal decapeptide with a decreased inhibition of human cathepsins B and L by three orders of magnitude [28]. We have used human CysC isolated from urine that is amino-terminally decapeptide truncated [41] and observed a low effect of CysC on the activity of cathepsin B. We have shown that the protective effect of this form of CysC is similar to that of recombinant full length human CysC and to a mutated recombinant full length CysC that lacks inhibitory activity [29]. These data preclude cathepsin B inhibition as the mechanism of protection by CysC.

We have used a range of assays in order to establish that CysC enhances autophagy in neuronal cells under toxic conditions as a protective mechanism. The combination of assays used showed that the observed increases in numbers of autophagosomes after exposure to CysC reflects induction of a fully functional autophagy pathway that includes competent proteolytic clearance of autophagy substrates by lysosomes. We first demonstrated that CysC protects neuronal cells from a variety of toxic insults, except for the toxicity induced by 3MA, an inhibitor of autophagy. We also showed that inhibition of autophagy by siRNA silencing of beclin 1 prevents CysC from protecting the cells against serum deprivation-induced death. We then showed that CysC causes redistribution of LC3 to vesicular profiles with increased levels of immunoreactive LC3-II vesicles accompanied by increased conversion of LC3-I to LC3-II, as identified by Western blotting. This change is associated with increased appearance of AVs in the cytoplasm of cells with otherwise normal ultrastructural morphology coupled with evidence for reduced mTOR activity (p70S6 kinase dephosphorylation). These observations strongly point to CysC-stimulated induction of autophagy in the absence of evidence for cellular degenerative changes (for review [42]). A final link of evidence indicating that increased autophagosome formation reflects increased autophagic activity is the demonstration by metabolic labeling that the rate of long-lived proteins breakdown was increased by CysC treatment under nutrition deprivation conditions. All these data combined indicate enhanced flux through a fully functional autophagy pathway, resulting in delivery to lysosomes where degradation occurs.

Autophagy is generally a cell survival mechanism [43], although sustained over-activity or dysfunction of the autophagic pathway mediates a caspase-independent form of cell death that shares certain features with apoptosis [31]. Autophagy appeared at an early stage of apoptosis of CA1 pyramidal neurons of the gerbil hippocampus after brief ischemia; however, and it was proposed that it protected the cells from death [42], [44]. Our data suggest that the increase in CysC immunoreactivity in the hippocampus of animals in response to injury (for review [3]) induces autophagy as an intrinsic neuroprotective mechanism.

There are several indications that CysC plays a protective role in AD, a neurodegenerative disorder characterized by deposition of Aβ in the brain. 1) Genetic data demonstrated linkage of the CysC gene (*CST3*) with an increased risk of developing late-onset AD (for review [3], [45]). The polymorphism in *CST3* results in reduced CysC secretion [46]–[48], and decreased CysC plasma levels [49], [50]. Mutations in the presenilin 2 gene linked to familial AD (PS2 M239I and T122R) alter CysC trafficking in mouse primary neurons causing reduced CysC secretion [51]. Reduced levels of CysC may represent the molecular factor responsible for the increased risk of AD and/or increased susceptibility to insult. 2) Immunohistochemical analyses have shown intensely CysC immunoreactive neurons and activated glia in the cerebral cortex of some aged human cases and of all AD patients [52], [53]. Neuronal staining of CysC in AD brains was primarily limited to pyramidal neurons in cortical layers III and V, which are the neurons most susceptible to cell death in AD [53], [54]. 3) Immunohistochemical studies revealed co-localization of CysC with Aβ amyloid deposits in brains of patients with AD, Down's syndrome, cerebral infarction [54]–[58] and non-demented aged individuals [54]. 4) CysC binds Aβ and inhibits Aβ oligomerization [59] and fibril formation *in vitro* [60] and *in vivo* [61], [62]. However, deletion of CysC in knockout mice resulted in an increase in cathepsin B activity and an enhanced Aβ degradation [63]. Unlike a complete deletion of CysC, reduced or enhanced levels of CysC expression affect the aggregation of Aβ, not Aβ levels [61], [62]. Extensive research has shown that Aβ has an important role in the pathogenesis of neuronal dysfunction in AD (for review [64]). Aβ has been shown to induce protein oxidation and lipid peroxidation both *in vitro* and *in vivo*, and was suggested to play a central role as a mediator of free radical induced oxidative stress and neurotoxicity in AD brain (for review [65]). We have demonstrated that the addition of CysC together with preformed oligomeric or fibrillar Aβ to either cultured primary hippocampal neurons or to N2a cells increased cell survival. While CysC inhibits Aβ aggregation, it does not dissolve preformed Aβ fibrils or oligomers [59], [60]. Thus, CysC has a dual protective effect from Aβ toxicity. It inhibits Aβ aggregation, in addition to the direct neuroprotective effect that is independent of its anti-Aβ amyloidogenetic property.

Autophagy has been implicated in the neurodegeneration and amyloidogenesis of AD [66], [67]. Extensive autophagic pathology observed in AD includes massive accumulation of AVs in affected neurons and dystrophic neurites and most likely arises from impaired clearance of AVs [68]. Aβ is normally generated by autophagic turnover of the amyloid precursor protein and subsequently cleared within the lysosomal system but builds up in accumulated AVs in AD brain [69]–[71]. Beclin 1 is reported to be decreased in AD, further disrupting autophagy and promoting Aβ generation [71]. A decrease in CysC levels in AD patients may contribute to this pathology. Our data show multiple possible neuropathological consequences of the reduced levels of CysC in AD, including an increase in Aβ accumulation and amyloid deposition as well as a reduced ability to protect from toxicity in the brain.

Accumulating data show that CysC functions via several independent pathways in response to a variety of toxic insults. The data presented here reveal a novel protective pathway that is independent of protease inhibition and involves induction of autophagy as a necessary aspect of the protective mechanism. Therapeutic manipulation of CysC levels resulting in slightly higher concentrations than physiological, either systemic or local, could protect neuronal cells from cell death in neurodegenerative disorders, such as Alzheimer's disease.

## Materials and Methods

### Cell culture

N2a cells (ATCC, Manassas, VA) were grown in Dulbeco's Modified Eagle Medium (DMEM) (Gibco Life Technologies-Invitrogen, Grand Island, NY) supplemented with 10% fetal bovine serum (FBS) (Gemini Bio-products, West Sacramento, CA), 1% penicilin-streptomicin, and 1% glutamine. For serum-deprivation studies cell cultures were washed twice with warm PBS and once with warm serum-free medium and incubated in either serum-supplemented or serum-free medium. Different concentrations of human urinary CysC (Calbiochem- EMD Bioscience, San Diego, CA) were added into the medium. The autophagy inhibitor, 3-methyladenine (3MA) (10 mM; Sigma, St Louis, MO), was added to serum-free medium in the presence or absence of CysC.

### Primary cultures of cortical neurons

Primary cultures of cortical neurons were established from either prenatal E19 pups of pregnant Sprague-Dawley rats (Charles River Labs), or from prenatal E16 pups of pregnant mice. All procedures involving experiments vertebrate animals received prior approval of the Nathan S. Kline Institute Animal Care and Use Committee, in accord with the provisions of the PHS “Guide for the Care and Use of Laboratory Animals” and the “Principles for the Utilization and Care of Vertebrate Animals”. Brains were placed in cold Hybernate E/A medium (BrainBits, Springfield, IL), and cortices containing the hippocampus were dissected out using a dissection microscope. Cortices were minced and incubated in 20 u/ml papain solution (Worthington) for 20 min at 37°C. The tissue was triturated in a papain blocking solution (20% FBS from Hyclone, 0.2 mg/ml DNase, and 0.1 M MgSO_4_ in neurobasal medium with supplements). Cell suspension was filtered through a 0.4 µm filter unit and centrifuged at 200×g for 3 min. The pellet containing the dissociated neurons was resuspended in neurobasal medium with supplements (2% B27, 100 U/ml penicillin, 100 µg/ml streptomycin sulfate and 0.30% glutamine) and viable cells were counted using a haemocytometer. Neurons were plated at a density of 2.5×10^6^ cells/cm^2^ on 50 µg/ml poly-d-lysine coated coverslips and incubated at 37°C in 5% CO_2_ atmosphere. Half of the culture medium was replaced every 3 days. Neuronal cultures were treated after 6–7 days in culture. For B27-supplement-deprivation studies neuronal cultures were extensively washed in saline solution I (8.1 g/l NaCl, 0.4 g/l KCl, 0.15 g/l Na_2_HPO_4_, 0.15 g/l KH_2_PO_4_, 4 g/l dextrose pH 7.2) and then incubated in B27-supplemented or -deprived neurobasal medium in the presence or absence of different concentrations of CysC. 10 µM H_2_O_2_, 0.5 µM colchicine, or 0.1 µM staurosporine were added into B27-supplemented neurobasal medium.

### Beclin 1 silencing

N2a cells were treated with 10 nM of *beclin 1* siRNA (Mm 214165 (a) or Mm 214172 (b); Qiagen, Valencia CA) and 20 nM Lipofectamine RNAiMAX (Invitrogen, Carlsbad, CA) in 20% Optimem (Gibco, Carlsbad, CA) and 80% complete medium (DMEM, Gibco, Carlsbad, CA) supplemented with 1% Glutamine (Gibco, Carlsbad, CA) and 10% fetal bovine serum (Cellgro, Manassas, VA) using a reverse transfection method in 96 well plate (200 µl final volume, 2500 cells per well) for 72 hours. Inhibition of beclin 1 expression was verified by Western blot analysis with anti-beclin 1 antibody (BD Transduction Bioscience, San Jose, CA). N2a cells were then washed twice with PBS, once with serum-free medium (DMEM (Gibco, Carlsbad, CA) supplemented with 1% Glutamine (Gibco, Carlsbad, CA) and incubated in serum-free medium with or without CysC (0.75 µM) for 48 hours. Mean and standard error of the mean (SEM) were calculated for 3 separate experiments.

### Neuronal viability assay by Hoechst staining

Cell cultures were washed with PBS, incubated with 0.4 µg/ml Hoechst 33258 (Invitrogen, Carlsbad, CA) solution for 5 min at room temperature, washed, fixed with 4% paraformaldehyde for 20 min at room temperature, and mounted using aqueous mounting medium with anti-fading agents (Biomeda, Foster City, CA). Live cells from entire wells were counted using the MCID's automatic target detection program in an Olympus BX60 system. Neuronal survival was expressed as percentage of neuronal survival in cultures incubated in serum-containing medium. Mean and standard error of the mean (SEM) were calculated for 4 separate experiments.

### CellTiter 96 Aqueous One Solution Cell Proliferation Assay (MTS)

20 µl of the CellTiter 96 Aqueous One Solution Reagent (Promega, Madison, WI) were added into each well of the 96 wells plate containing cells in 100 µl culture medium, incubated with the reagent for 3 hours at 37°C in 5% CO_2_ and absorbance at 490 nm was recorded. As a negative control cell-free medium was included. Neuronal survival was expressed as percentage of neuronal survival in serum-free medium cultures. Mean and SEM were calculated for 3 separate experiments.

### Production and isolation of CysC variants

Intact, full-length, CysC and an anti-proteolytically defect CysC-variant with the residues 8, 9, 10 and 106 in its inhibitory center replaced by glycine-residues were isolated from periplasmic extracts of bacterial clones containing the appropriate expression plasmids as previously described [29]. CysC, devoid of the 10 amino-terminal residues of protein, resulting in reduced antiproteolytic activity, was isolated from native human urine as earlier described [72].

### Immunohistochemical staining for LC3-II

Cell cultures were washed with PBS and fixed in 4% paraformaldehyde in PBS for 20 min at room temperature. After PBS washing, cells were blocked for 1 hour at room temperature with 2% BSA, 2% FBS in PBS, and incubated with anti-LC3-II antibody (1∶500) in blocking solution containing 0.1% saponin overnight at 4°C. Cells were incubated in Alexa Fluor 488 goat anti-rabbit IgG (1∶100, Invitrogen, Carlsbad, CA) for 2 hours at room temperature, mounted with aqueous gel mount (Biomeda, Foster City, CA), and visualized by a fluorescence microscope (Olympus BX60).

### Western blot analysis

Cells were harvested in lysis buffer (1% Nonidet P-40, 1% Na deoxycolate, 0.1% SDS, 150 mM NaCl, 10 mM Na phosphate pH 7.2) containing protease inhibitors (5 µg/ml Leupeptin-Antipain-Pepstatin A mix, 1 mM PMSF) and 1% phosphatase inhibitors for phosphorylated proteins and centrifuged at 10,000×g for 10 min. Protein concentration was determined by BCA Protein Assay Kit (Pierce, Rockford, IL). Equal amounts (10–30 µg) of total proteins were boiled in sample buffer (1% SDS, 3% glycerol, 1.5% β-mercaptoethanol and 20 mM Tris-HCl, pH 6.8) and separated by 4–12% tris-glycine gel electrophoresis. The proteins were electrophoretically transferred onto a 0.2 µm nitrocellulose membranes (BioRad, Hercules, CA) in 2.5 mM Tris/19.2 mM Glycine/20% methanol transfer buffer. The membranes were blocked in 5% milk (BioRad, Hercules, CA) or 5% BSA in 10 mM Tris, 150 mM sodium chloride, pH 7.5, 0.1% Tween-20 (TBST), incubated with primary antibody for 2 hours at room temperature or overnight at 4°C, and with secondary antibody for 1–2 hours at room temperature, and in chemiluminiscent fluid (Millipore, Billerica, MA) for 60 seconds prior to exposure. The membranes were imaged using a Fuji LAS-3000 gel documentation unit for 10–60 seconds. Quantification was performed by digital image using the native Fuji software, ImageGauge. For LC3, 20 µg was loaded per lane onto 16% tris-gylcine gels, and following electrophoresis and transfer, membranes were incubated for 4 hours with the primary antibody in 3% milk with TBST, 1 hour in secondary antibody, and in chemiluminescent fluid (Millipore, Billerica, MA) [68], [70], visualized on Reflection Autoradiography film and bands intensity was quantified using the Quantity One program. Mean and SEM were calculated for 4 separate experiments.

*Antibodies used:* anti-rat LC3 + anti-human LC3 (1∶1,000), anti-P70S6 kinase (1∶1,000; Cell Signaling, Danvers, MA), anti-p-P70S6 kinase (1∶1,000; Cell Signaling, Danvers, MA), beclin 1 (1∶1,500; BD Transduction Bioscience, San Jose, CA), β-tubulin (Pierce, Rockford, IL), β-actin (Abcam, Cambridge, MA).

### Electron Microscopy

Cells incubated on coverslips were washed thrice in either serum-free DMEM or supplement-free Neurobasal media, and fixed in 4% paraformaldehyde and 1% glutaraldehyde in 0.1 mol/L sodium cacodylate buffer (pH 7.2) overnight at room temperature. Following fixation, coverslips containing cells were washed in cacodylate buffer, postfixed in 1% osmium tetroxide, and progressively dehydrated through graded series of ethanol. The cells were then *in situ* embedded following infiltration in embedding medium (Epon 812 Mixture; Electronmicroscopy Sciences) by inverting 2/3 filled beem (size # 3) capsules over the cells. Thin sections were cut, mounted onto copper grids, contrasted with Uranyl acetate and Reynold's lead citrate, and examined using Philips CM 10 electron microscope. Images were captured on a digital camera (Hamamatsu; model C4742-95) using Advantage CCD Camera System software (Advanced Microscopy Techniques Corporation). The number of AVs *per* cell was counted for at least 20 cells *per* condition, in images taken at magnitude of×7,900 (64 µm^2^). Mean and SEM results of three independent experiments are presented. Types of AVs were identified by visual inspection of the micrographs using previously established criteria [13], [66]. Briefly, AVs (vesicles >0.5 µm) were classified as autophagosomes when they met two or more of the following criteria: double membranes (complete or at least partially visible), absence of ribosomes attached to the cytosolic side of the membrane, luminal density similar to cytosol, and identifiable organelles or regions of organelles in their lumen. Vesicles of similar size but with a single membrane (or less than 40% of the membrane visible as double), luminal density lower than the surrounding cytosol, multiple single membrane-limited vesicles containing light or dense amorphous material were classified as autophagolysosomes.

### Cathepsins B activity assay

Cells in 100 mm plates were scraped in 5 ml of PBS, centrifuged, washed with PBS, and homogenized at pH 7.0. 30% of the sample was used for Western blot analysis. Homogenates were acidified at pH 5.5 and Cathepsin B was assayed by measuring the release of 7-amino-4-methylcoumarin (amc) from Z-Arg-Arg-amc as described [73]. Assays were performed in white micro plates in a volume of 200 µl mixture (1–5 µl of enzyme or lysate; 50 µl of 0.1% Brij-35; and 145 µl of 0.1 M sodium phosphate buffer pH 6.0 containing 2 mM EDTA and DTT; 1 mM PMSF, 5 µM pepstatin A and 5 mM Z-Arg-Arg-amc). Fluorescence of amc released after two hours was read in a Wallac Victor-2 spectrofluorimetric plate reader with a filter set optimized for detection of 4-methyl-7-aminocoumarin (-*amc*) standard solution with excitation at 365 nm and emission at 440 nm. Enzyme activity was expressed as the amount of amc released per hour per mg protein.

### Intracellular Protein Degradation Measurements

Total protein degradation in cultured cells was measured by pulse-chase experiments [30]. Confluent cells were labeled with [^3^H]-leucine (2 µCi/ml) for 48 hours at 37°C in order to preferentially label long-lived proteins. Following labeling, cells were extensively washed and maintained in complete medium (DMEM + 10% FBS), under which conditions autophagy is suppressed, or in serum-deprived medium, where autophagy is induced. Under both conditions, after washing the cells, the medium was supplemented with unlabeled 2.8 mM leucine to prevent [^3^H]-leucine reutilization. Aliquots of the medium taken at different time-points were precipitated with 10% TCA, filtered using a 0.22 µm pore membrane and radioactivity in the flow-through and in the filters was measured. Proteolysis was expressed as the percentage of the initial acid-precipitable radioactivity (protein) transformed to acid-soluble radioactivity (amino acids and small peptides) over time. Where indicated, either 20 mM NH_4_Cl and 100 µM leupeptine or 10 mM 3MA were added immediately after the labeling period and maintained at that concentration throughout the chase.

### Statistical analyses

The statistical significance was determined by one-way analysis of variance (ANOVA) followed by the Bonferroni *post hoc* test to determine significance of differences between multiple test groups. Student's T-test was used to determine significance when only two groups were compared. Differences were significant for *p = 0.05; **p = 0.01; and ***p = 0.001.
